# Cloning and Functional Characterization of Dihydroflavonol 4-Reductase Gene Involved in Anthocyanin Biosynthesis of Chrysanthemum

**DOI:** 10.3390/ijms21217960

**Published:** 2020-10-27

**Authors:** Sun-Hyung Lim, Bora Park, Da-Hye Kim, Sangkyu Park, Ju-Hee Yang, Jae-A Jung, JeMin Lee, Jong-Yeol Lee

**Affiliations:** 1 Division of Horticultural Biotechnology, School of Biotechnology, Hankyong National University, Anseong 17579, Korea; 2 National Institute of Agricultural Sciences, Rural Development Administration, Jeonju 54874, Korea; pbr0915@naver.com (B.P.); kimdh143@korea.kr (D.-H.K.); psk2779@korea.kr (S.P.); juheeppuing@korea.kr (J.-H.Y.); 3 Department of Horticultural Science, Kyungpook National University, Daegu 41566, Korea; jemin@knu.ac.kr; 4 Floriculture Research Division, National Institute of Horticultural & Herbal Science, Rural Development Administration, Wanju 55365, Korea; jabisung@korea.kr

**Keywords:** anthocyanin, chrysanthemum, dihydroflavonol 4-reductase, flavonoid, flower color

## Abstract

Dihydroflavonol 4-reductase (DFR) catalyzes a committed step in anthocyanin and proanthocyanidin biosynthesis by reducing dihydroflavonols to leucoanthocyanidins. However, the role of this enzyme in determining flower color in the economically important crop chrysanthemum (*Chrysanthemum morifolium* Ramat.) is unknown. Here, we isolated cDNAs encoding DFR from two chrysanthemum cultivars, the white-flowered chrysanthemum “OhBlang” (CmDFR-OB) and the red-flowered chrysanthemum “RedMarble” (CmDFR-RM) and identified variations in the C-terminus between the two sequences. An enzyme assay using recombinant proteins revealed that both enzymes catalyzed the reduction of dihydroflavonol substrates, but CmDFR-OB showed significantly reduced DFR activity for dihydrokaempferol (DHK) substrate as compared with CmDFR-RM. Transcript levels of anthocyanin biosynthetic genes were consistent with the anthocyanin contents at different flower developmental stages of both cultivars. The *in* *planta* complementation assay, using *Arabidopsis thaliana dfr* mutant (*tt3-1*), revealed that *CmDFR-RM,* but not *CmDFR-OB,* transgenes restored defective anthocyanin biosynthesis of this mutant at the seedling stage, as well as proanthocyanidin biosynthesis in the seed. The difference in the flower color of two chrysanthemums can be explained by the C-terminal variation of CmDFR combined with the loss of *CmF3H* expression during flower development.

## 1. Introduction

**Figure 1:**
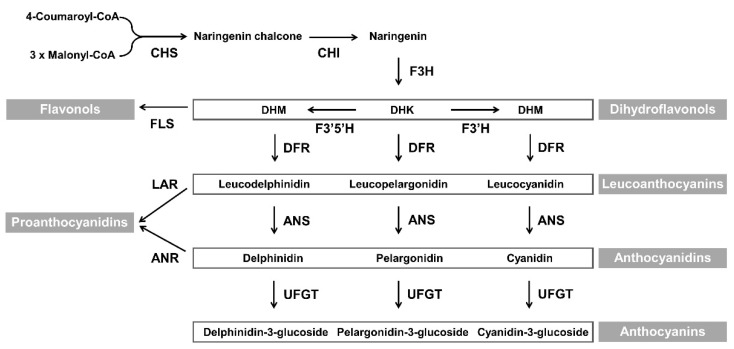
Schematic representation of flavonols, proanthocyanidins, and anthocyanins biosynthetic pathway in plants The abbreviations of enzyme names are as follows: CHS, chalcone synthase; CHI, chalcone isomerase; F3H, flavanone 3-hydroxylase; FLS, flavonol synthase; F3′H, flavonoid 3′-hydroxylase; F3′5′H, flavonoid 3′,5′-hydroxylase; DFR, dihydroflavonol 4-reductase; LAR, leucoanthocyanidin reductase; ANR, anthocyanidin reductase; ANS, anthocyanidin synthase; and UFGT, UDP-glucose: flavonoid 3-*O*-glucosyltransferase.

Flower color influences the commercial value of ornamental plants. In general, floral coloration involves the accumulation of pigments such as flavonoids (including anthocyanins), carotenoids, and betalains. The flavonoid class anthocyanins are responsible for flower colors ranging from pale pink to blue in many ornamental plants including chrysanthemum (*Chrysanthemum morifolium*), carnation (*Dianthus caryophyllus*), lily (*Lilium longiflorum*), gerbera (*Gerbera hybrida*), petunia (*Petunia hybrida*), and rose (*Rosa hybrida*). The mechanism of anthocyanin biosynthesis has been extensively investigated, and most structural and regulatory genes involved in this process have been identified in several plant species [1,2,3]. Dihydroflavonol 4-reductase (DFR) plays an important role in the formation of anthocyanins and proanthocyanidins, as shown in the Figure 1. DFR catalyzes the conversion of the colorless dihydroflavonols, including dihydrokaempferol (DHK), dihydroquercetin (DHQ), and dihydromyricetin (DHM), into leucoanthocyanidins. These three colorless dihydroflavonols are substrates of flavonol biosynthesis that are catalyzed by flavonol synthase (FLS). Competition between FLS and DFR can modify the metabolic flux and alter flower color [4,5,6].

Several studies have revealed that the substrate specificity of DFR plays an important role in the determination of anthocyanin types [7,8,9,10]. According to differences at the 134th amino acid residues in their substrate binding domains, DFRs are divided into three types, the asparagine (Asn)-type, aspartic acid (Asp)-type, and non-Asn/Asp-type. Previous studies have reported that Asn-type DFRs catalyze the conversion of all three dihydroflavonols (DHK, DHQ, and DHM), whereas Asp-type DFRs are highly specific for DHQ and DHM rather than for DHK as a substrate [8,9,10]. However, not all Asn-type DFRs can catalyze all three dihydroflavonols as substrates. DFRs from sweet potato (IbDFR) and from freesia (FeDFR2) are classified into the Asn-type DFR, due to their 134th amino acid residues [10,11]. IbDFR catalyzes only the DHK but not DHQ and DHM, while FeDFR2 utilizes both the DHQ and DHM but not the DHK as a substrate. Other studies have shown that the neighboring residues of the substrate binding sites and NADP binding regions of DFRs play pivotal roles in protein activity in buckwheat (*Fagopyrum esculentum*), gerbera, and grape hyacinth (*Muscari armeniacum*) [11,12,13]. These results imply that other factors can influence the substrate specificity of DFR enzyme.

Chrysanthemum, a perennial herbaceous flowering plant, is an important floricultural crop worldwide. The ray florets of chrysanthemum flowers can be pink, red, yellow, white, purple, green, or various bicolored forms due to the accumulation of different proportions of two pigments, i.e., anthocyanins and carotenoids [14]. There are three basic types of anthocyanidins, namely, pelargonidin, cyanidin, and delphinidin. Although the ray florets of chrysanthemum only accumulate the cyanidin derivatives, the substrate feeding assay revealed that CmDFR could utilize DHK and DHM as a substrate for pelargonidin and delphinidin biosynthesis, respectively [14,15,16].

In the current study, we identified two DFR genes, *CmDFR-OB* and *CmDFR-RM,* with different C-terminus, from chrysanthemum flowers with white and red colors, respectively, and demonstrated that these genes function in anthocyanin biosynthesis. We performed an in vitro enzyme assay with recombinant CmDFRs and verified their catalytic activities for different dihydroflavonol substrates. In a complementation assay with the *Arabidopsis thaliana* mutant *transparent testa3-1* (*tt3-1*), *CmDFR-RM* restored anthocyanin accumulation in rosette leaves and proanthocyanidin accumulation in the seeds, but *CmDFR-OB* did not. Our study indicates that the CmDFRs play a functional role in anthocyanin biosynthesis and pigmentation in the ray florets of chrysanthemum.

## 2. Results

### 2.1. Nucleotide Polymorphisms of the CmDFR Gene in Different Colored Ray Florets Chrysanthemum Cultivars

**Figure 2:**
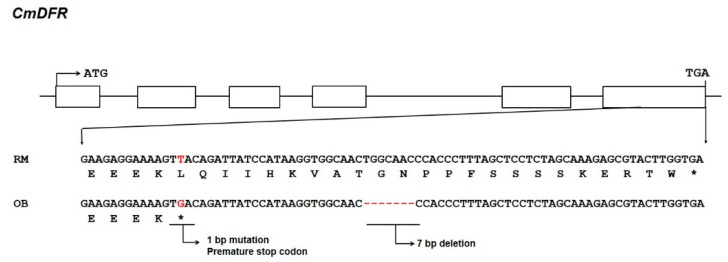
Schematic representation and comparison of the nucleotide sequences of *DFR* genes in chrysanthemum cultivars OhBlang (OB) and RedMarble (RM). Exons are indicated by open boxes and introns by a thin line. Different sequence regions are marked with a dashed red line, and the asterisk indicates a stop codon.

The NCBI database lists four full-length *DFR* clones from chrysanthemum. All of these genes share at least 95% sequence similarity with the first *DFR* isolated from chrysanthemum *CmDFR* (accession ADC96612.1), a 1125 bp sequence with an ORF encoding a deduced protein of 374 amino acids. To investigate the role of DFR in anthocyanin biosynthesis, we isolated *DFR* sequences from OhBlang (OB) and RedMarble (RM) using primers designed based on the *CmDFR* sequences. Unexpectedly, the cDNA sequences from OB and RM showed a variation in the C-terminal region as compared with ADC96612.1. Thus, we designated *CmDFR* from OB as *CmDFR-OB*, a 1059 bp sequence with an ORF encoding a deduced protein of 352 amino acids (GenBank accession number MT513763). *CmDFR* from RM (*CmDFR-RM*) is 1128 bp long, with an ORF encoding a 375 amino acid protein (GenBank accession number MT513764). The high level of amino acid sequence identity among CmDFR, CmDFR-OB, and CmDFR-RM suggests that the genes encoding these proteins are allelic. Multiple sequence alignment of DFRs from chrysanthemum revealed a single nucleotide substitution (T→G) that resulted in a premature stop codon, and also a 7 bp deletion within the sixth exon of *CmDFR-OB* (Figure 2).

**Figure 3:**
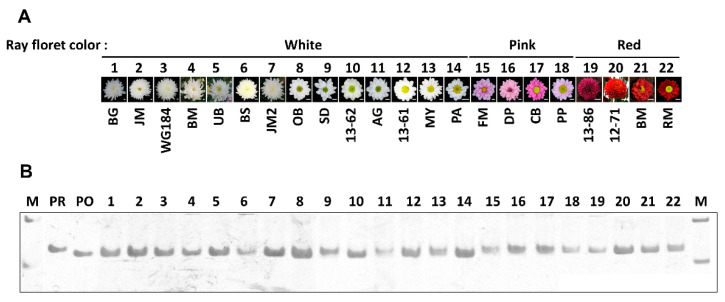
Insertion and deletion (InDel) analysis of the *CmDFR* gene in chrysanthemum. (**A**) Photographs of 22 chrysanthemum flowers showing different ray floret colors, scale bar indicates 1 cm; (**B**) PCR products amplified using InDel-DFR primers and separated on an 6% polyacrylamide gel. M, DNA molecular weight markers. PR and PO used as InDel marker control represent the amplified fragment from the positive plasmids containing *CmDFR-RM* and *CmDFR-RM*, respectively.

According to the nucleotide polymorphism of *CmDFR*, we screened 22 chrysanthemum cultivars (14 white, 4 pink, and 4 red) using an insertion and deletion (InDel) maker (Figure 3). To validate the accuracy of the InDel marker, we used plasmids containing *CmDFR-OB* and *CmDFR-RM* as positive controls for analysis. Consistent with the results of DNA sequencing analysis, the *CmDFR* fragment amplified from RM was 127 bp in size and that from OB was 120 bp in size due to a 7 bp deletion (Figure 3B). PCR using InDel marker generated 120 bp amplicons from all 11 chrysanthemum cultivars with white ray florets except for AG, MY, and SD. By contrast, 127 bp amplicons were produced from all eight chrysanthemum cultivars with pink or red colored ray florets and in the positive control *CmDFR-RM*. These results suggest that that the *DFR* gene alone cannot be used to determine the ray floret color.

**Figure 4:**
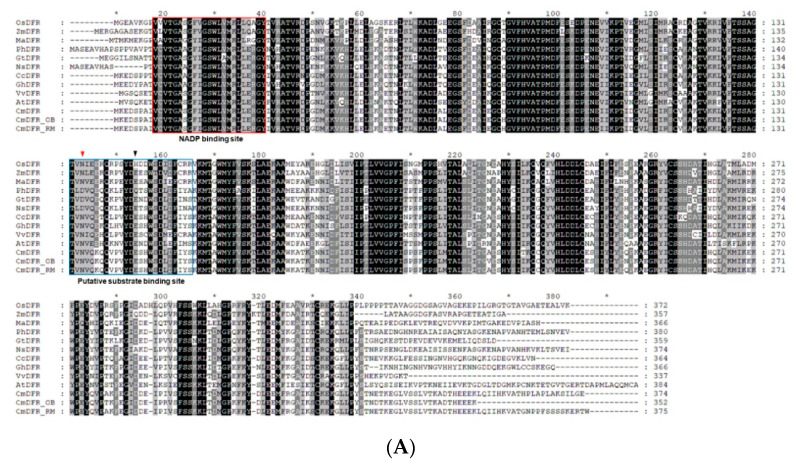

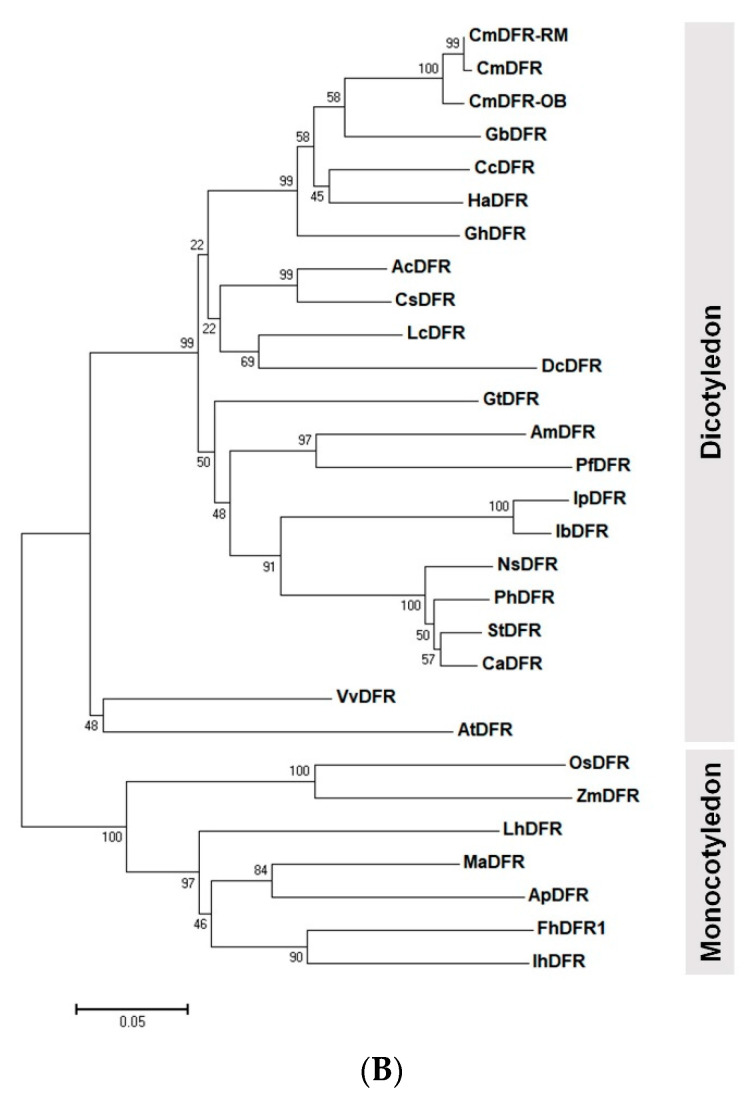
Amino acid sequence alignment and phylogenetic analysis of DFR proteins in *Chrysanthemum morifolium* and those from other species. (**A**) Multiple sequence alignment of CmDFRs and DFR proteins from other species. Numbers indicate the position of the last amino acid in each protein sequence. The putative NADP binding site and presumed substrate binding region are marked by blue and red boxes, respectively. Substrate specificity is associated with aa134 and aa145 (highlighted with an inverted red arrowhead and an inverted black arrowhead, respectively); (**B**) Phylogenetic relationships among DFR proteins from chrysanthemum and other plants. The phylogenetic tree was constructed using the neighbor-joining method with MEGA6 software. Numbers next to the nodes are bootstrap values from 1000 replications. The tree is drawn to scale and has branch lengths with the same units as those of the evolutionary distances that were used to infer the phylogenetic tree (scale bar, 0.05 amino acid substitutions per site). The GenBank accession numbers of the protein sequences used are as follows: *Actinidia chinensis* AcDFR (PSS36490.1); *Agapanthus praecox* ApDFR (AB099529.1); *Antirrhinum majus* AmDFR (X15536); *Arabidopsis thaliana* AtDFR (AB033294); *Callistephus chinensis* CcDFR (P51103.1); *Camellia sinensis* CsDFR (AAT84073.1); *Capsicum annuum* CaDFR (NP_001311706.1); *Chrysanthemum morifolium* CmDFR (ADC96612), CmDFR-OB (MT513763), CmDFR-RM (MT513764); *Daucus carota* DcDFR (XP_017254990.1); *Freesia hybrida* FhDFR1 (KU132393); *Gentiana triflora* GtDFR (BAA12736.1); *Gerbera hybrida* GhDFR (AKN56969.1); *Gynura bicolor* GbDFR (BAJ17657.1); *Helianthus annuus* HaDFR (XP_022001438.1); *Ipomoea batatas* IbDFR (BAD05164.1); *Ipomoea purpurea* IpDFR (BAA36406.1); *Iris × hollandica* IhDFR (BAF93856.1); *Lilium × hybrida* LhDFR (BAB40789.1); *Lonicera caerulea* LcDFR (ALU09329.1); *Muscari armeniacum* MaDFR (AIC33028.1); *Nierembergia* sp. NB17 NsDFR (BAC10993.1); *Oryza sativa*, OsDFR (AB003495); *Perilla frutescens* PfDFR (AB002817); *Petunia hybrida* PhDFR (CAA56160); *Solanum tuberosum* StDFR (AEN83503.1); *Vitis vinifera* VvDFR (X75964); *and Zea mays* ZmDFR (Y16040).

Sequence alignment showed that CmDFRs contain a highly conserved NADP binding region and a substrate binding region at their N-termini, whereas their C-termini are diverse (Figure 4A). These conserved NADP binding domains and substrate binding domains are present in dicot plants such as Arabidopsis, cupflower (*Nierembergia spathulata*), gentian (*Gentiana triflora*), gerbera, grape (*Vitis vinifera*), and petunia, and also in monocot species such as grape hyacinth, rice (*Oryza sativa*), and maize (*Zea mays*), suggesting that these proteins are highly conserved among angiosperms.

Phylogenetic analysis of DFRs from various plant species clearly classified DFRs from monocots and eudicots into different branches (Figure 4B). DFR proteins including CmDFR, CmDFR-OB, and CmDFR-RM were grouped into the same subclade with gerbera in the Asteraceae family. Similar to the DFRs of the Asteraceae family, petunia PhDFR was classified close to potato StDFR, pepper CsDFR, and cupflower NsDFR in the Solanaceae family. The results of phylogenetic analysis align well with the genetic relationships among the species. Collectively, our data suggests that these chrysanthemum DFRs possessing the conservative characteristics can have a functional DFR role in flavonoid biosynthesis.

### 2.2. Recombinant CmDFRs Exhibit Different Enzymatic Properties

**Figure 5:**
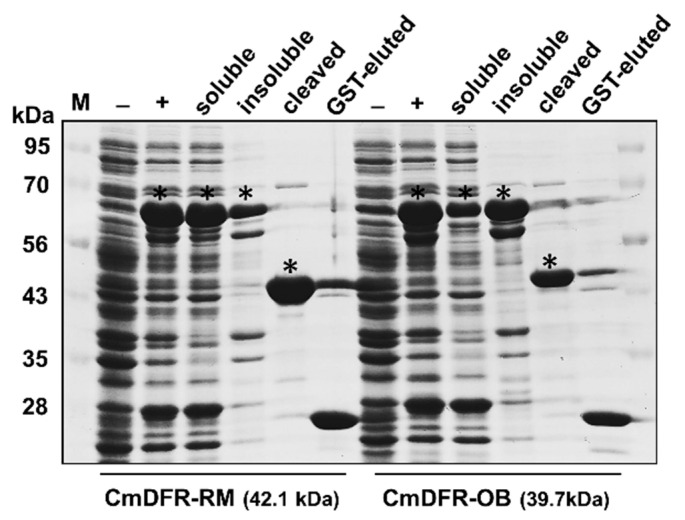
Expression and purification of recombinant CmDFR proteins. The coding regions of *CmDFRs* from RM and OB without the stop codon were cloned into pGEX-6P-1 in-frame with a glutathione *S*-transferase (GST) tag and expressed in *Escherichia coli* BL21 (DE3) cells. Bacterial lysates were prepared for gel loading before (–) and after (+) IPTG induction. The IPTG-induced cells were lysed by sonication and separated into the aqueous fraction (soluble) and non-aqueous pellet (insoluble) by centrifugation. Recombinant proteins in the aqueous fraction were collected using Glutathione Sepharose 4B beads, and their GST tag was removed by PreScission protease (cleaved). The GST moiety that remained on the beads was eluted (GST-eluted). The samples were subjected to SDS-PAGE to confirm the expression and purification of the proteins. Asterisks indicate induced recombinant CmDFR proteins displayed at corresponding size with or without GST moiety.

We assayed the enzyme activity of recombinant CmDFR proteins in vitro. We successfully produced two types of recombinant CmDFRs (CmDFR-OB, and CmDFR-RM) in IPTG-treated transformed bacterial cells. The majority of glutathione *S*-transferase (GST)-CmDFR-RM was detected in the soluble fraction, whereas GST-CmDFR-OB was mostly partitioned into the insoluble inclusion body (Figure 5). Although a relatively small amount of CmDFR-OB was obtained as compared with CmDFR-RM due to their reduced solubility, both GST-cleaved proteins were successfully purified.

**Figure 6:**
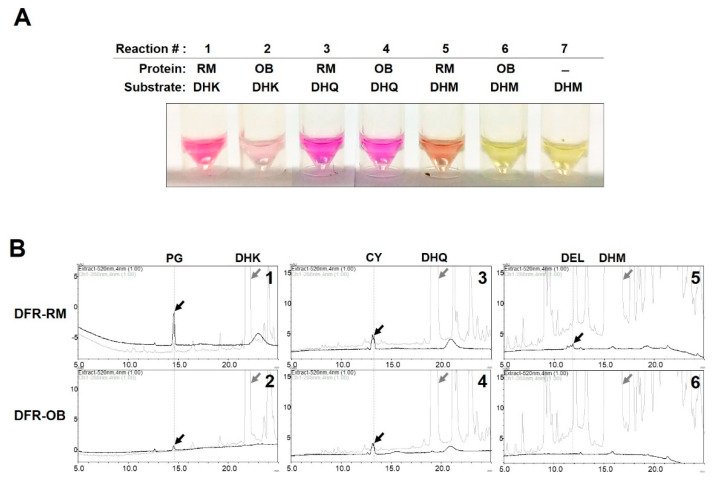
In vitro assays of recombinant CmDFR proteins. (**A**) DFR activities of recombinant CmDFR-OB, and CmDFR-RM using the dihydroflavonol substrates DHK, DHQ, and DHM. The unstable leucoanthocyanidins produced by DFR activity were chemically converted to stable anthocyanidins, and the corresponding color development was observed; (**B**) HPLC analysis of anthocyanidin metabolites generated by the enzyme activity of CmDFR-OB, and CmDFR-RM. Black and grey chromatograms and arrows indicate anthocyanidins production and dihydroflavonols substrates detected at 520 and 288 nm, respectively. PG, pelargonidin; CY, cyanidin; DEL, delphinidin.

We performed and in vitro enzyme activity assay using dihydroflavonol substrates and equal amounts of the two recombinant CmDFR proteins. Since the products generated from these reactions, leucoanthocyanidins, were unstable, we chemically converted these compounds to stable anthocyanidins with acidic alcohol and identified the corresponding anthocyanidins based on specific color development and HPLC analysis. Both CmDFRs converted DHK to leucopelargonidin, as identified by color development (Figure 6A) and peaks corresponding to pelargonidin in HPLC (Figure 6B). In this reaction, CmDFR-RM exhibited high enzymatic activity, whereas CmDFR-OB showed only slight activity. When DHQ was used as the substrate, both CmDFRs showed comparable levels of activity, although CmDFR-OB activity was somewhat lower than CmDFR-RM. The catalytic activity for the substrate dihydromyricetin (DHM) was detected only in CmDFR-RM. When extracting the reactant with ethyl acetate, the recovery rate of each leucoanthocyanidin product varies depending on their B-ring hydroxylation [7]. Therefore, the difference in substrate preference between each dihydroflavonol substrate could not be determined from the results of this experiment, but these results clearly showed that the catalytic activity of CmDFR-OB was lower than that of CmDFR-RM, and in particular, its activity for DHK substrate was drastically decreased.

### 2.3. Anthocyanin Content and Expression Analysis of CmDFR in OB and RM

**Figure 7:**
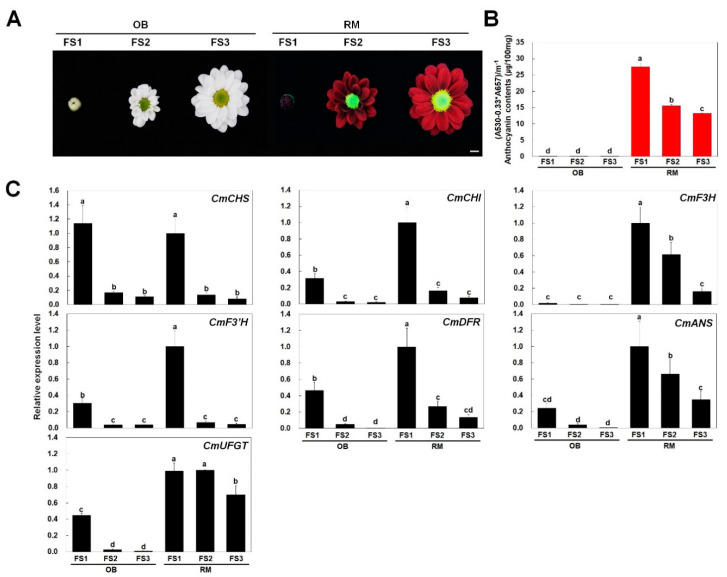
Phenotypes, anthocyanin contents, and anthocyanin biosynthetic gene expression levels in chrysanthemum cultivars OB and RM. (**A**) Photographs of flowers at different developmental stages (FS1, FS2, and FS3) from the two chrysanthemum cultivars examined in this study; (**B**) Anthocyanin contents in ray florets of both cultivars; (**C**) qPCR analysis of anthocyanin biosynthetic genes including four early biosynthetic genes (chalcone synthase (*CHS),* chalcone isomerase *(CHI),* flavanone 3-hydroxylase (*F3H)*, and flavonoid 3′-hydroxylase (*F3*′*H)*), and three late biosynthetic genes (dihydroflavonol 4-reductase (*DFR)*, anthocyanidin synthase (*ANS),* and UDP-glucose: flavonoid 3-*O*-glucosyltransferase (*UFGT)*). Results represent mean values ± SD from three biological replicates. *CmEF1α* was used as the reference gene. Different letters above the bars indicate significantly different values (*p* < 0.05) calculated using one-way ANOVA followed by a Duncan’s multiple range tests.

To investigate the relationship between pigmentation and anthocyanin content, we measured the anthocyanin contents in ray florets at three developmental stages of the white cultivar OB and the red cultivar RM (Figure 7A). The anthocyanin contents were consistent with the visible appearance of the ray florets of RM (Figure 7B). In these plants, the anthocyanin content was highest at flowering stage 1 (FS1) and gradually decreased during flower development. These results indicate that variations in anthocyanin content are responsible for the color differences in ray florets of OB vs. RM.

To investigate the relationship between color phenotype and transcript levels of anthocyanin biosynthetic genes, we analyzed the expression of seven structural genes including four early biosynthetic genes (EBG), i.e., chalcone synthase (*CHS*), chalcone isomerase (*CHI*), flavanone 3-hydroxylase (*F3H*), and flavonid 3′-hydroxylase (*F3*′*H*), and three late biosynthetic genes (LBG), i.e., dihydroflavonol 4-reductase (*DFR*), anthocyanidin synthase (*ANS*), and UDP-glucose: flavonoid 3-O-glucosyltransferase (*UFGT*) by quantitative reverse-transcription PCR (qPCR) (Figure 7C). For both OB and RM, transcript level of EBG was highly detected at FS1 and dramatically decreased at FS2. Except for *CmCHS*, all of EBG was highly detected in RM as compared with OB. Interestingly, transcript level of *CmF3H* was barely detected in all flower developmental stage of OB, while it was highly expressed in those in RM. In the expression profile of LBG, OB showed a high transcript level at FS1, but a sharply low transcript level according to the flower development. However, RM showed high expression pattern at FS1 and gradually reduced transcript level until FS3. Overall, it showed that coordinated expression of anthocyanin biosynthetic genes, except of *CmCHS* coincided with the patterns of anthocyanin accumulation at different stages of flower development.

### 2.4. CmDFR-RM Complements the Arabidopsis tt3-1 Mutant

**Figure 8:**
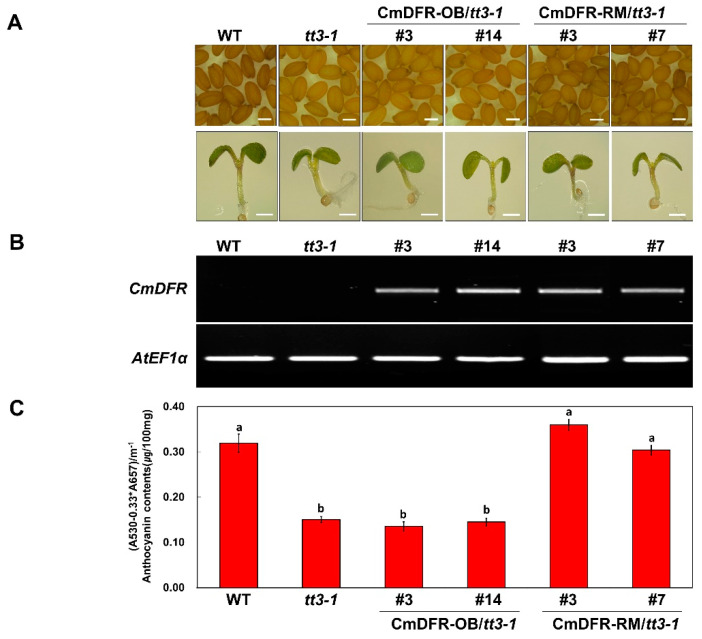
Overexpression of *CmDFR-OB* and *CmDFR-RM* in Arabidopsis *tt3-1* mutants. (**A**) Seeds (top) and 3-day-old seedlings (bottom) of wild-type Col-0, *tt3-1*, and representative T_2_ progeny of homozygous Arabidopsis *tt3-1* lines transformed with *CmDFR-OB* and *CmDFR-RM*, respectively. White bar indicates 0.5 mm in size; (**B**) Expression analysis of *CmDFR* in the leaves of wild-type, *tt3-1*, and transgenic plants by RT-PCR. *AtEF1α* was used as the reference gene; (**C**) Anthocyanin contents of four-week-old Arabidopsis plants. Results represent mean values ± SD from three biological replicates. Different letters above the bars indicate significantly different values (*p* < 0.0001) calculated using one-way ANOVA followed by a Duncan’s multiple range tests.

We evaluated the effects of *CmDFR-OB* and *CmDFR-RM* on anthocyanin biosynthesis by introducing these genes into the Arabidopsis *tt3-1* mutant, which failed to accumulate brown proanthocyanidins in seeds and anthocyanins in the junction between the stem and rosette leaves. We obtained transgenic *CmDFR-OB* and *CmDFR-RM* plants via the floral dip method and selected transformants by spraying the plants with 0.3% basta. To investigate the role of the CmDFRs in anthocyanin accumulation, wild-type seeds, mutant, and T_2_ transgenic lines were germinated and grown on 1/2 Murashige and Skoog (MS) medium containing 1% sucrose (Figure 8A). Transformation with *CmDFR-OB* failed to restore the mutant phenotypes of *tt3-1*, including a lack of proanthocyanidins in seeds and anthocyanins at the junction of the cotyledon and hypocotyl. However, transformation with *CmDFR-RM* restored these mutant phenotypes, as the transgenic plants accumulated proanthocyanidins in seeds and anthocyanins at the junction of the cotyledon and hypocotyl.

At the mature stages, we performed the RT-PCR to check the presence and expression of the *CmDFR* gene (Figure 8B). It revealed that the *CmDFR* gene was successfully expressed in all the transgenic Arabidopsis plants. Furthermore, we extracted pigments from whole leaves of wild-type, *tt3-1*, and *tt3-1* plants complemented with *CmDFRs* (Figure 8C). The anthocyanin content of *tt3-1* was half that of wild-type plants. The anthocyanin contents of complemented *tt3-1* plants harboring *CmDFR-RM* were similar to that of the wild-type plants. By contrast, the anthocyanin content of *tt3-1* plants complemented with *CmDFR-OB* was not restored. These results suggest that CmDFR-RM functions in proanthocyanidins biosynthesis in seeds and anthocyanins biosynthesis in leaves and stems.

## 3. Discussion

DFR is a NADPH-dependent reductase that converts dihydroflavonols such as DHK, DHQ, and DHM into leucoanthocyanidins such as leucopelargonidin, leucocyanidin, and leucodelphinidin, respectively. DFR proteins contain the highly conserved NADP(H) binding domain “VTGAAGFIGSWLIMRLLERGY” and a substrate binding domain. The latter domain is divided into three types including the Asn type, Asp type, and non-Asn/Asp type, which differ in the amino acid residue at the 134th position. Due to the different preferences of DFRs for dihydroflavonols, these enzymes affect the biosynthesis of anthocyanin metabolites. Asn-type DFRs can use all three dihydroflavonols (DHK, DHQ, and DKM) as substrates, whereas Asp-type DFRs cannot use DHK efficiently [8,9,10]. For example, as Asp-type DFRs from petunia and cymbidium cannot catalyze the conversion of DHK efficiently, they fail to produce brick red flowers in nature [8,9].

DFR is the key flavonoid biosynthetic enzyme contributing to the accumulation of pigments including anthocyanin and proanthocyanidin. In this study, we purified the two recombinant CmDFRs from bacterial expression (Figure 5) and demonstrated their DFR activities (Figure 6). During the process of protein purification, unlike the recombinant CmDFR-OB, the majority of recombinant CmDFR-RM protein was detected in the soluble fraction, which appeared to be associated with the C-terminal region of CmDFR-RM containing multiple hydrophilic residues (-SSSSKERT-). Although it cannot be ascertained whether these variations at the C-terminus of the two DFRs directly or indirectly affect the enzyme activity, at least the C-terminal hydrophilic region appears to affect the solubility of DFR protein.

CmDFR-OB and CmDFR-RM both contain the 134th Asn residue, which enable the catalysis of all three dihydroflavonol substrates including DHK. The result of in vitro assay showed that both enzymes produced leucopelargonidin and leucocyanidin from DHK and DHQ, respectively. Leucodelphinidin production was very scare or absent both of CmDFR-OB and CmDFR-RM, but it needs to expand its detection range to confirm. The enzymes both showed similar activities for leucocyanidin production. Interestingly, there was a striking difference in the production of leucopelargonidin. CmDFR-OB showed a dramatically reduced activity as compared with CmDFR-RM on leucopelargonidin production. This remarkable decrease in the activity of CmDFR-OB can be assumed to be due to the C-terminal truncation, suggesting that the variable C-terminus of DFR may be an important region conferring structural stability of binding pocket, especially near the B-ring binding site. A recent study showed that MaDFR harboring a point mutation at the 134th or 145th residue was still able to catalyze reactions using all three dihydroflavonol substrates [17], which indicated that the 134th or 145th residue was not an absolute factor for substrate specificity of DFR. Therefore, it can be implied that the C-terminal variable region of DFR may be one of the important factors that determine substrate specificity of DFR.

Differences in the C-terminal region of DFR proteins have been associated with different substrate preferences and enzyme activities [10,18]. In poplar, two DFR proteins, PtrDFR1 and PtrDFR2, belonging to the Asp type DFRs, exhibited the variability at the C-terminus. The tobacco flower color change, due to anthocyanin accumulation, was observed in the ectopic expression of PtrDFR1, but not in that of PtrDFR2. In freesia, two DFR proteins (FeDFR1 and FeDFR2) classified into the same Asn type with high sequence similarity, but they exhibited the C-terminal variation each other. In vitro enzyme assay showed that FeDFR2 was able to convert DHQ into leucocyanidin, while FeDFR1 had no catalytic activity on DHQ. Taken together these results suggest that the variable C-terminus of DFR plays an important role in substrate preference and enzyme activity.

Several studies have indicated that anthocyanin biosynthesis takes place within a metabolon complex consisting of CHS, F3H, F3′H, DFR, and ANS, in which DFR might interact with ER-bounded cytochrome P450 to direct the metabolic flux towards anthocyanin biosynthesis [19,20]. In the metabolon complex, the F3′H and flavonoid 3′,5′-hydroxylase (F3′5′H) enzymes catalyze B ring hydroxylation. The sequential interaction between F3′H and DFR or between F3′5′H and DFR results in the accumulation of cyanidin- and delphinidin-derived anthocyanins, respectively. Additionally, substrate channeling between DFR and FLS for anthocyanin and flavonol biosynthesis contributes to color pattern formation in flowers [4,21].

**Figure 9:**
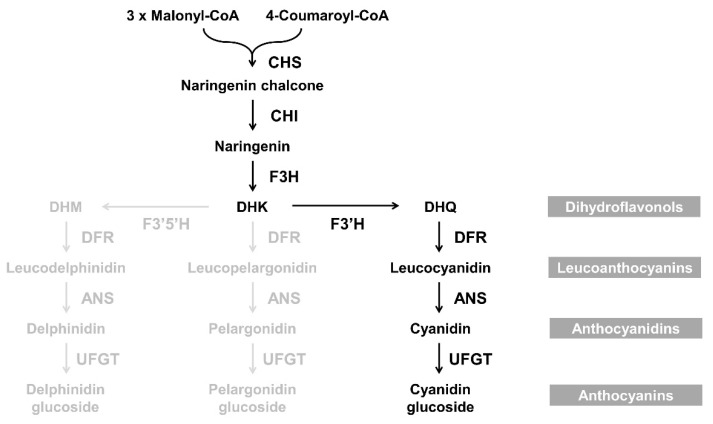
The proposed anthocyanin biosynthetic pathway in the ray florets of chrysanthemum. The bold black lines indicate the biosynthetic pathway of cyanidin-based anthocyanins that are only accumulated in the ray florets of Chrysanthemum flowers. The abbreviations of enzyme names are as follows: CHS, chalcone synthase; CHI, chalcone isomerase; F3H, flavanone 3-hydroxylase; FLS, flavonol synthase; F3′H, flavonoid 3′-hydroxylase; F3′5′H, flavonoid 3′,5′-hydroxylase, DFR, dihydroflavonol 4-reductase; ANS, anthocyanidin synthase; and UFGT, UDP-glucose: flavonoid 3-*O*-glucosyltransferase.

Several reports have indicated that ray florets of chrysanthemum with bronze, pink, or purplish-red coloration accumulate only cyanidin derivatives, whereas white ray florets do not accumulate any anthocyanins [14,15,22]. In the current study, we detected anthocyanins in the red-flowered cultivar RM but not in the white-flowered cultivar OB, indicating that anthocyanins were responsible for the coloration of these flowers. Additionally, we analyzed the expression of anthocyanin biosynthetic genes in ray floret of OB and RM to better understand anthocyanin biosynthetic mechanism in chrysanthemum (Figure 7). Our study showed that most flavonoid biosynthetic genes, except for *CmCHS*, were expressed at dramatically low levels in all floral developmental stage of OB as compared with that in RM. Interestingly, the transcript level of *CmF3H* among EBG was hardly detected in OB during all flower developmental stages. Although all of LBG at FS1 in OB was expressed at a low level, it was not able to accumulate anthocyanin in the ray florets of OB. As well as, we verified that CmDFR-OB can catalyze the DHK and DHQ as substrates with in vitro assay (Figure 6). Taken together these results suggest that the lack of *CmF3H* transcripts may lead to the deficient of DHK and block off the metabolic flow toward anthocyanin biosynthesis in OB. Additionally, it is thought that the high activities of F3′H can fully convert DHK into DHQ, resulting in the predominant accumulation of B-ring dihydroxylated metabolites, which lead to accumulate cyanidin-based anthocyanins and quercetin derivatives [14,15]. On the basis of these results, we propose the anthocyanin biosynthetic pathway in ray florets of chrysanthemum, as shown in Figure 9.

The *in planta* assay confirmed that the ectopic expression of *CmDFR-RM* restored the phenotype of Arabidopsis *tt3-1* to wild type, and therefore this mutant accumulated proanthocyanidin in its seeds and anthocyanin in its hypocotyls and mature leaves. However, the ectopic expression of *CmDFR-OB* did not restore the accumulation of proanthocyanidin and anthocyanin to wild-type levels. In wild-type Arabidopsis seedling, cyanidin and pelargonidin accumulate at comparable levels and the major flavonol is kaempferol, [23] indicating that Arabidopsis F3′H capacity only accepts a fraction of the total flavonoid metabolites. Therefore, the failure of *tt3-1* restoration by CmDFR-OB suggests that pelargonidin, which accounts for about half of anthocyanins, was barely produced in the transgenic lines due to dramatically decreased DHK acceptance of CmDFR-OB.

Taken together, our results indicate that the difference of DFR enzyme activity and lack of *CmF3H* transcripts may contribute to the anthocyanin accumulation of chrysanthemum. Further studies on the interactions between metabolon enzyme complex in chrysanthemum would provide additional insights into anthocyanin biosynthesis in the ray florets of this crop.

## 4. Materials and Methods

### 4.1. Plant Materials

Chrysanthemum plants were grown in a greenhouse under short day conditions at the National Institute of Horticultural and Herbal Science (Wanju, Korea). Two chrysanthemum cultivars named as OhBlang (OB) with white flower and RedMarble (RM) with red flower, respectively were used to analyze anthocyanin levels and gene expression. For the InDel marker analysis, we used twenty-two chrysanthemum cultivars. These cultivars are categorized based on their ray floret color as follows: white (BaekGang (BG), JimMa (JM), WonGyo 184 (WG184), BaekMa (BM), UnBaek (UB), BaekSeol (BS), JimMa2 (JM2), OB, SnowDream (SD), 13-62, AnGel (AG), 13-61, MoYa (MY), and PureAngel (PA)); pink (FreeMadona (FM); DonaPink (DP), CherryBlossom (CB), and PinkPride (PP)); and red (13-86, 12-71, BlackMarble (BM), and RM).

Transformation experiments were conducted using *Arabidopsis thaliana* transparent testa (*tt*) *dfr* mutant line *tt3-1*, which was obtained from the Arabidopsis Biological Resource Center (ABRC). All Arabidopsis plants were grown on 1/2 MS medium containing 1% sucrose or in soil under long-day conditions (LD, 16 h light/8 h dark) at 22 °C.

All samples were frozen rapidly in liquid nitrogen and kept at −80 °C. A portion of the samples was used for RNA extractions and anthocyanin measurements.

### 4.2. CmDFR Gene Cloning and Sequence Analysis

The full-length ORF (open reading frame) of *CmDFR* was identified from the chrysanthemum genome database based on *Chrysanthemum boreale* and was predicted using FGENESH (http://www.softberry.com/berry.phtml?topic=fgenesh&group=programs&subgroup=gfind).

Genomic DNA was obtained from chrysanthemum leaves using a DNeasy Plant Mini Kit (Qiagen, Valencia, CA, USA), according to the manufacturer’s instructions. Total RNA was extracted from ray florets of flowers at different developmental stages from two different chrysanthemum cultivars OB and RM using Fruit-mate for RNA Purification solution (Takara, Otsu, Japan) and Plant RNA Purification Reagent (Invitrogen, Carlsbad, CA, USA), as described previously [24]. The total RNA was purified using a FavorPrep™ Plant Total RNA Mini Kit (Favorgen, Changzhi, Taiwan), according to the manufacturer’s instructions. cDNA was synthesized from 2 μg of total RNA using amfiRivert cDNA Synthesis Platinum Master Mix (GenDEPOT, Barker, TX, USA). The *CmDFR* gene was amplified from cDNA and genomic DNA by PCR with PrimeSTAR^®^ HS DNA Polymerase (Takara) and the primer set CmDFR-F/R. All PCR fragments were subcloned into the pENTR/D-TOPO vector (Invitrogen) to validate the DNA sequences. All primer sequences are listed in Supplementary Table S1.

Nucleotide sequence, deduced amino acid sequence, and ORF of *CmDFR* were determined online (http://www.ncbi.nlm.nih.gov). Structural analysis of the deduced protein was carried out using the ExPASy Molecular Biology Server (http://cn.expasy.org/tools/). Multiple sequence alignments were performed using the CLUSTALW program (https://www.genome.jp/tools-bin/clustalw). A phylogenetic tree was constructed using the neighbor-joining method [25] with MEGA version 6 software.

### 4.3. InDel Analysis

Genomic DNA was extracted from the leaves of the 22 chrysanthemum cultivars using a DNeasy Plant Mini Kit (Qiagen), following the manufacturer’s instructions. A pair of PCR primers was designed to detect the 7-bp deletion in the sixth exon of *CmDFR* (Supplementary Figure S1). The PCR mixture contained 100 ng of genomic DNA, 5 pmol of each primer, 10 pmol of dNTPs, and 1 unit of PrimeSTAR HS DNA Polymerase in 1 × PrimeSTAR Buffer (Takara) in a total volume of 25 μL. The PCR conditions were as follows: An initial denaturation at 98 °C for 2 min, followed by 30 cycles of denaturation at 98 °C for 10 s, annealing at 60 °C for 10 s, and an extension at 72 °C for 20 s, and a final incubation at 72 °C for 5 min. The PCR products were separated in a 5% nondenaturing polyacrylamide gel in 1 × TBE buffer (90 mM Tris-borate, 2 mM EDTA, pH 8.0). Following electrophoresis, the DNA fragments were visualized by silver staining, as previously described [26].

### 4.4. Expression and Purification of Recombinant CmDFR

The *CmDFR* ORFs were amplified using primer sets designed as shown in Supplementary Table S1. The PCR products were inserted into the pGEX-6P-1 vector linearized by *Bam*HI digestion using an In-Fusion Advantage PCR Cloning Kit (Clontech, Mountain View, CA, USA) in-frame with the sequence encoding the N-terminal glutathione *S*-transferase (GST) tag. The resulting pGEX-6P-1:*CmDFR* vectors were verified by sequencing and transformed into *Escherichia coli* strain BL21 (DE3) cells (Novagen, Darmstadt, Germany). Transformed bacterial cells were cultured in 50 mL of LB broth containing 50 μg·mL^–1^ ampicillin. Protein expression was induced by the addition of 0.1 mM isopropyl *β*-*D*-1-thiogalactopyranoside (IPTG) at 20 °C for 20 h. After centrifugation, the collected bacterial cells were lysed by sonication in 2 mL of sonication buffer containing 50 mM sodium phosphate (pH 8.0), 150 mM NaCl, 1% Triton X-100, 10% glycerol, 2 mM dithiothreitol (DTT), and 1 mM phenylmethylsulfonyl fluoride. Following centrifugation (13,000× *g*, 4 °C, 10 min), 100 μL of Glutathione Sepharose 4B beads (GE Healthcare, Pittsburgh, PA, USA) were added to the soluble bacterial lysate and incubated at 4 °C for 2 h, with gentle rotation. The GST-tagged protein-bound beads were collected and washed five times with 1 × PBS (137 mM NaCl, 2.7 mM KCl, 100 mM Na_2_HPO_4_, and 2 mM K_2_HPO_4_ (pH 7.4)). For GST cleavage, a mixture of 4 μL of PreScission protease (GenScript, Piscataway, NJ) and 96 μL of cleavage buffer (50 mM Tris-HCl (pH 7.0), 150 mM NaCl, 1 mM EDTA, and 1 mM DTT) was added to the beads and incubated for 3 h, at 4 °C. Following incubation, the beads were pelleted by centrifugation at 500× *g* for 3 min and the eluate was transferred to a new tube. The beads were washed with two bed volume of cleavage buffer, and the washing solution was combined with the eluate. The concentrations of the GST-cleaved CmDFR proteins were equalized to 1.1 μg/μL by adding cleavage buffer, and the same volume of glycerol was added to obtain a final concentration of 0.55 μg/μL. These were used for assay and the rest were stored in −20 °C. The purified untagged proteins in the eluate were quantified by the Bradford method and verified by SDS-PAGE.

### 4.5. In Vitro Assay for CmDFR Activity

The enzymatic activities of the CmDFRs were assayed in a 200 μL reaction containing 3.3 μg of GST-cleaved CmDFR protein, 100 mM Tris-HCl (pH 7.5), 1 mM glucose 6-phosphate, 0.75 mM nicotinamide adenine dinucleotide phosphate reduced, 1 unit of glucose 6-phosphaste dehydrogenase, and 1 mM of dihydroflavonol substrates. The reactions were initiated by adding dihydroflavonol substrates and incubated at 30 °C for 50 min, and 10 μL acetic acid was added to stop the reaction. The reactants were extracted twice with 500 μL of ethyl acetate and dried using nitrogen gas. The products of the reactions were unstable leucoanthocyanidins; therefore, the residues were dissolved in 100 μL of acidic alcohol, *n*-butanol:HCl (95:5, *v*/*v*) to generate the corresponding stable anthocyanidins. Following incubation at 95 °C for 30 min, 10 μL of the solution was analyzed by high-performance liquid chromatography (HPLC).

### 4.6. HPLC Analysis

HPLC analysis was conducted on an LC-20A HPLC system with a diode array detector (Shimadzu, Kyoto, Japan). The separation of dihydroflavonol substrates and anthocyanidin products was accomplished on an Inertsil-ODS3 C18 column (5 *μ*m, 250 × 4.6 mm, GL Science). The mobile phase consisted of 0.1% (*v*/*v*) formic acid (A) and acetonitrile containing 0.1% (*v*/*v*) formic acid (B). The gradient profile was optimized as follows: 0 min, 95% A/5% B; 30 min, 45% A/55% B; 45 min, 35% A/65% B; 50 min, 0% A/100% B; 52 min, 95% A/5% B; and 60 min, 95% A/5% B. The flow rate was 1 mL·min^−1^, and the column temperature was maintained at 30 °C. The detection wavelength was 288 nm for dihydroflavonols and 520 nm for anthocyanidins.

### 4.7. Chemical Standards

(±)-DHK, (±)-DHQ, and (±)-DHM were purchased from Sigma-Aldrich (St. Louis, MO, USA), and pelargonidin chloride, cyanidin chloride, and delphinidin chloride were purchased from Extrasynthese (Extrasynthese, Genay Cedex, France). The dihydroflavonols were prepared as 100 mM stock solutions in DMSO, and the anthocyanidins were prepared as a 100 mM solution in 50% methanol containing 1.2 N HCl.

### 4.8. Measurement of Total Anthocyanin Contents

Total anthocyanin contents were measured from ray florets of flowers at the following different developmental stages: FS1, 6.0 weeks under short-day (SD) treatment, on which the first coloration of ray florets was first visible; FS2, 7.0 week under SD treatment that is the date of early flowering; and FS3, 7.5 weeks under SD treatment that is the date of full flowering. As described by [24], powdered ray floret samples were incubated in 600 μL extraction buffer (methanol containing 1% HCl) for 6 h, at 4 °C, with moderate shaking. Following the addition of 200 μL water and 200 μL chloroform, the samples were centrifuged at 14,000× *g* for 5 min at 4 °C to sediment the plant material. The absorbance of the supernatant was recorded at 530 nm (A_530_) and 657 nm (A_657_) using a microplate reader. Anthocyanin content was determined using the following equation: A_530_ − 0.33 × A_657_. The anthocyanin content in each sample was measured in three independent experiments.

### 4.9. Quantitative Reverse-Transcription PCR (qPCR)

Total RNA was prepared from ray florets, as described above. cDNA was synthesized from 2 μg of total RNA using amfiRivert cDNA Synthesis Platinum Master Mix (GenDEPOT).

qPCR was performed using AccuPower 2x Greenstar qPCR Master Mix (Bioneer, Daejun, Korea) and a Bio-Rad CFX96 Detection System (Bio-Rad Laboratories, Hercules, CA, USA), according to the manufacturer’s instructions. Gene expression was normalized using the elongation factor 1α (*EF1α*) as the reference gene. The gene-specific primers used for qPCR analysis are listed in Supplementary Table S1. Three biological replicates were performed per sample.

### 4.10. In Planta Assay of CmDFR Function

The plasmid used for stable transformation of Arabidopsis was constructed as follows: The ORFs of *CmDFR* isolated from cultivars OB (*CmDFR-OB*) and RM (*CmDFR-RM*) were subcloned into the pENTR/D-TOPO vector (Invitrogen) and incorporated into the Gateway destination vector pB7WG2D (VIB-Ghent University, Ghent, Belgium) via several Gateway cloning steps. The resulting vector was maintained in *Agrobacterium tumefaciens* strain GV3101 and transformed into the Arabidopsis *tt3-1* mutant using the floral dip method. Transformed Arabidopsis seeds were grown in soil under LD conditions at 22 °C. Transgenic Arabidopsis plants were selected by spraying the plants with 0.3% basta solution. Homozygous T_2_ lines were subjected to evaluate expression level of exogenous *CmDFR* gene and phenotypic investigation. *Arabidopsis elongation factor 1α* (*EF1α*) gene was used as internal reference.

### 4.11. Statistical Analysis

For qPCR analysis, results were represented as mean values ± SD from three biological replicates. For the analysis of anthocyanin contents, experimental data were presented as mean values ± SD of three biological replicates. Statistical significance was determined by one-way ANOVA followed by a Duncan’s multiple range tests.

## 5. Conclusions

In this study, we characterized the DFR derived from different ray floret colored OB and RM chrysanthemum cultivars. *CmDFR2-OB* contains a premature stop codon due to a single base substitution. Differences at the C-terminus of CmDFR proteins affect their catalytic efficiency, as well as protein solubility. Lack of *CmF3H* gene expression was observed in OB during all flower developmental stages. Complementation tests indicated that the C-terminal variation in CmDFR also affects anthocyanin levels in leaves and proanthocyanidin biosynthesis in seeds, as revealed in transgenic Arabidopsis. Taken together, these results provide a new insight to better understand the anthocyanin biosynthesis in chrysanthemum.

## Supplementary Materials

Supplementary Materials can be found at https://www.mdpi.com/1422-0067/21/21/7960/s1, Table S1: Primer sequences used in this study.

## Abbreviations

ANS	Anthocyanidin synthase
CHI	Chalcone isomerase
CHS	Chalcone synthase
DFR	Dihydroflavonol 4-reductase
F3H	Flavanone 3-hydroxylase
F3′H	Flavonoid 3′-hydroxylase
F3′5′H	Flavonoid 3′5′-hydroxylase
FLS	Flavonol synthase
InDel marker	Insertion and deletion marker
qPCR	Quantitative reverse-transcription PCR
RT-PCR	Reverse-transcription PCR
UFGT	UDP-glucose: flavonoid-3-O-glucosyltransferase.
