# Supplementary material for: Cloning and Functional Characterization of Dihydroflavonol 4-Reductase Gene Involved in Anthocyanin Biosynthesis of Chrysanthemum
Source: Int J Mol Sci. 2020 Oct 27;21(21):7960. doi: 10.3390/ijms21217960 (PMC7663526; doi:10.3390/ijms21217960)
Supplement: Supplementary file 1 [file ijms-21-07960-s001.pdf]

Supplementary Table 1. Primers sequences used in this study.

Usage	Primer name	Primer sequence
Gene expression	qRT-CmDFR-F	GCAGCATGGAAAGCAACAAAG
	qRT-CmDFR-R	GGGACTGATAAATGGACCAACAAC
	qRT-CmEF1α-F	TTTTGGTATCTGGTCCTGGAG
	qRT-CmEF1α-R	CCATTCAAGCGACAGACTCA
Gene cloning	CmDFR-F	GGAGTTGAATGGCGGATGCAACACGCACGT
	CmDFR-R	GTCCATAGGGGTGGCAACATGAAACACTC
Indel analysis	Indel-DmDFR-F	GGAGGGACTCGTCTCATCTC
	Indel-DmDFR-R	TCACCAAGTACGCTCTTTGC
In vitro enzyme activity	pGEX6p1-CmDFR-F	GGGGCCCCTGGGATCCATGAAAGAAGACTCAC
	pGEX6p1-CmDFR-R	GGAATTCCGGGGATCCTTATTCACCAAGTACGC
	pGEX6p1-CmDFR-OB-R	GGAATTCCGGGGATCCTCACTTTTCCTCTTCG
	pGEX6p1-CmDFR-RM-R	GGAATTCCGGGGATCCTCACCAAGTACGCTCT
Gene expression	qRT-CmCHS-F	CAAGGAGGAGAAGATGAGAG
	qRT-CmCHS-R	CCGAACCCGAATAAAACAC
	qRT-CmCHI-F	GAACCATTTTGATGAGAAAGCA
	qRT-CmCHI-R	TTGCACCCTTAAACTCCTGTT
	qRT-CmF3H-F	AGCGAAGCCCATTGAAAGTA
	qRT-CmF3H-R	TGGCAAGAACACACGCTAAC
	qRT-CmF3'H-F	AGGCGGATTCATCGTTTC
	qRT-CmF3'H-R	ACTCTTTGGGCTTATCAGG
	qRT-CmDFR-F	GCAGCATGGAAAGCAACAAAG
	qRT-CmDFR-R	GGGACTGATAAATGGACCAACAAC

	qRT-CmANS-F	AGGATTAGGACTTGAGGAGGGG
	qRT-CmANS-R	GGTTGAGGGCATTTTGGGTAG
	qRT-CmUFGT-F	TTCCCTTTTGCCTCACACCC
	qRT-CmUFGT-R	TTAAGAACCCTGCGAAACTCCT
	qRT-CmEF1α-F	TTTTGGTATCTGGTCCTGGAG
	qRT-CmEF1α-R	CCATTCAAGCGACAGACTCA
Transgene expression	RT-CmDFR-F	CATTGGCGGAGAAAGCAGCATGGAAA
	RT-CmDFR-R	GTGTCCGCCTTTGTTACTAGAGAT
	RT-AtEF1α-F	GCCACACCTCTCACATTG
	RT-AtEF1α-R	TACCAGCGTCACCATTCT
